# Silicon fertilizer mediated structural variation and niche differentiation in the rhizosphere and endosphere bacterial microbiome and metabolites of sugarcane

**DOI:** 10.3389/fmicb.2022.1009505

**Published:** 2022-09-29

**Authors:** Zhaonian Yuan, Ziqin Pang, Nyumah Fallah, Yongmei Zhou, Fei Dong, Wenxiong Lin, Chaohua Hu

**Affiliations:** ^1^Key Laboratory of Sugarcane Biology and Genetic Breeding, Ministry of Agriculture, Fujian Agriculture and Forestry University, Fuzhou, China; ^2^College of Agricultural, Fujian Agriculture and Forestry University, Fuzhou, China; ^3^Province and Ministry Co-sponsored Collaborative Innovation Center of Sugar Industry, Nanning, China; ^4^Center for Genomics and Biotechnology, Fujian Agriculture and Forestry University, Fuzhou, China; ^5^College of Life Sciences, Fujian Agriculture and Forestry University, Fuzhou, China

**Keywords:** sugarcane, compartment niches, co-occurrence networks, silicon fertilization practices, plant-microbe interactions, soil-plant continuum

## Abstract

The microbiomes of plant are potential determinants of plant growth, productivity, and health. They provide plants with a plethora of functional capacities, namely, phytopathogens suppression, access to low-abundance nutrients, and resistance to environmental stressors. However, a comprehensive insight into the structural compositions of the bacterial abundance, diversity, richness, and function colonizing various microenvironments of plants, and specifically their association with bioactive compounds and soil edaphic factors under silicon (Si) amendment remains largely inconclusive. Here, high-throughput sequencing technology and nontargeted metabolite profiling method were adopted to test the hypotheses regarding microbiome niche abundance, diversity, richness, function, and their association with bioactive compounds and soil edaphic factors within different ecological niches (leaf, stem, root, rhizosphere, and bulk soils) under Si amendment during cane growth were we addressed. Our results demonstrated that Si correspondingly increased sugarcane theoretical production and yield, and remarkably enhanced soil nutrient status, especially Si, AP, and AK. It was also observed that bacterial diversity demonstrated tissue-dependent distribution patterns, with the bulk soil, rhizosphere soil, and root endosphere revealing the highest amount of bacterial diversity compared with the stem and leaf tissues. Moreover, Si exhibited the advantage of considerably promoting bacterial abundance in the various plant compartments. Co-occurrence interactions demonstrated that Si application has the potential to increase bacterial diversity maintenance, coexistence, and plant–soil systems bacteria connections, thereby increasing the functional diversity in the various plant tissues, which, in turn, could trigger positive growth effects in plants. Network analysis further revealed that metabolite profiles exhibited a strong association with bacterial community structures. It was also revealed that Si content had a considerable positive association with bacterial structures. Our findings suggest that the dynamic changes in microbe’s community composition in different plant and soil compartments were compartment-specific. Our study provides comprehensive empirical evidence of the significance of Si in agriculture and illuminated on differential metabolite profiles and soil microbe’s relationship.

## Introduction

Plant rhizosphere has been widely regarded as a hot spot for different microorganisms and is known as one of the complex ecosystems. A great number of microbes, namely, bacteria and fungi, colonize rhizosphere soil and these microbes play a crucial role in both soil fertility and health, and plant growth and development (Hayat et al., 2010). Plant root systems secrete a series of organic secretions that can promote rhizospheric microbe’s growth (Walker et al., 2003), thereby forming a community of microorganisms in a rhizosphere microenvironment. The variations in plant root system rhizosphere deposition and secretion of plant species tend to influence rhizosphere bacterial abundance and community distribution patterns (Lamb et al., 2011; Lundberg et al., 2012). There is clear evidence that a vast majority of these rhizosphere microbes are associated with a complex food web that may use a significant portion of nutrients produced by the plants, thereby influencing their abundance and community distribution pattern (Mendes et al., 2013). In a study conducted by Cordero et al. (2019), it was demonstrated that bacterial abundance and composition in the wheat and canola rhizosphere compartments exhibited distinct distribution patterns, potentially driven by *Acidobacteria*, *Gemmatimonadetes,* and *Firmicutes*. They further postulated that these microbes were crop and organ-specific. Similarly, Ling et al. (2022) revealed that phyla Bacteroidetes and Proteobacteria peaked significantly in the rhizosphere soil, which was largely due to the fact that these bacteria are predominantly adapted to C-rich environment (e.g., rhizosphere soil) to enhance fast growth, propagation, and metabolic activity. Mounting evidence has also suggested that different plant compartments, namely, plant roots are colonized by various microbes. These microbes are capable of enhancing plant resistance to abiotic and biotic stresses, and can play a valuable role in agriculture husbandry. It is, therefore, crucial to have a better insight into how plant root systems influence microbes composition communities and diversity in plant root systems.

Plants endospheres are generally colonized by diverse microbes communities, which inhabit the plant internally during their growth and development and are characterized as endophytes (Giri and Dudeja, 2013; Dudeja et al., 2021). A number of microbial tend to enter plant roots system as endophytes and establish a mutual relationship. Endophytes can not only promote the growth and development of host plants, but also enhance the plants resistance through antagonism induced by pathogenic bacteria (Afzal et al., 2019; Dubey et al., 2020). Additionally, endophytes can promote plants to synthesize a number of plant growth hormones, enhance host plants ability to absorb soil nutrients, promote the growth and development of host roots and absorb various inorganic ions through a synthesis of iron carriers and nitrogen-fixing bacteria. Changes in root rhizosphere deposition and secretion and plant species could influence the distribution pattern of microbes abundance, community, and functional diversity in rhizosphere soil (Dennis et al., 2010), soil fertility (Yang et al., 2022), and plant growth and development (Shen et al., 2018). Moreover, a vast majority of these bacteria can carry genes that facilitate biological nitrogen fixation, possibly enabling them to transform dinitrogen gas (N_2_) into forms that are available to plant, namely, nitrate and ammonium within the occupied plant. Therefore, it is crucial to have a broader insight into the endophytes-plant relationship, which is vital to enhancing crop productivity.

Generally, different plant tissues can harbor a diverse group of microbial, and these microbes are component-specific (Wu et al., 2020). For instance, Beckers et al. (2017) observed distinct alterations in bacteria in the different plant tissues (soil, roots, stems, and leaves), and each part had specific bacterial composition. Their finding also revealed that the bacterial diversity diminished with the selection of plants, specifically the bacterial diversity and abundance in the rhizosphere zone were more pronounced than those in the plant. Similarly, Xiong et al. (2020) evaluated bacterial communities of 684 samples from rhizosphere and bulk soils, rhizoplane, root endosphere, phylloplane, and leaf endosphere in maize, wheat, and barley rotation system under soil amendments at two different sites. It was evident that microbiome assembly along the soil–plant continuum was altered primarily by host species and compartment niche compared with soil amendments or sites.

Silicon (Si) is widely deemed as the second richest element in the soil and earth’s crust. A large number of studies have demonstrated that Si is capable of promoting plant growth and enhancing plant-insect and disease resistance, and cold and drought resistance. On the other hand, the vast majority of Si in soil environments exists in the form of precipitates or silicate crystals, and the concentration of Si in soil solutions is usually limited. Moreover, the amount of plant-available Si in soils is deficient, hence utilizing Si properly can increase plant resistance against abiotic and biotic stresses, thereby improving soil health and fertility (Matychenkov and Ammosova, 1996), boosting crop growth and increasing crop yield (Meyer and Keeping, 2000). Despite these findings, our understanding of microbial community composition and diversity in multiple compartment niches (root, stem, leaf, rhizosphere soil, and non-rhizosphere) is still limited, especially under fertilization practices, such as Si amendment. Therefore, this study took sugarcane (ZZ6) as the material to evaluate the changes of bacterial community composition, diversity, and richness in the root, stem, leaf, rhizosphere soil, and non-rhizosphere soils of sugarcane after Si application, combined with the previous metabolomic data. This study aims to answer the following questions: (i) What is the effect of Si fertilizer on soil physiological, bacterial community, diversity, richness, and function during sugarcane growth? (ii) Does Si application has the potential to enhance the association among soil properties, core metabolites, and bacteria in the various soil and plant niches, and the co-occurrence network in plant–soil systems?

## Materials and methods

### Experimental design and sample collection

This study was conducted in Qumeng Village, Fusui County, Chongzuo City, Guangxi Province, China (22^o^49′N, 107^o^76′E). The area has an annual average temperature of 21.7°C and a rainfall of 1,121 mm. The basic soil properties of the site were assessed, including pH (4.22), organic matter (20.07 g/kg), total nitrogen (1.43 g/kg), total phosphorus (1.25 g/kg), and total potassium (1.18 g/kg). The field experimental plot was laid out in a randomized block design consisting of two treatments, and three replicates. The entire field contained 6 plots, with a total area of 1,800 m^2^ (6 m × 50 m × 6 plots). In mid-March 2019, rotary tillage was used to plow the soil (30 cm depth). Subsequently, sugarcane variety ZZ6 obtained from Guangxi University was cultivated at the rate of approximately 83,333/hm^2^, followed by fertilizer application. On each plot, the line between sugarcane was 1.2 and 0.1 m row spacing. The experiment consisted of two treatments, namely, (1) control, CK: compound fertilizer (NPK151515), and (2) silicon fertilizer, Si (NPK18-18-18 + Sikg/hm^2^). Si fertilizer was applied uniformly once, while the compound fertilizer was applied in different doses, and at two intervals. The first dose (40%) and second dose (60%) were applied during the seedling stage and elongation stage, respectively. The compound fertilizer was applied at the rate of 1,500 kg/hm^2^, while Si was applied at the rate of 1,250 kg/hm^2^. Si fertilizer contained SiO_2_ content greater than 10%. These fertilizers were obtained from Hebei Silicon Valley Academy of Agricultural Sciences.

### Sampling and preparation of samples

Samples of the rhizosphere soil, roots, stems, and leaves were collected on 28th November 2019, following the approach adopted by Beckers et al. (2017). Briefly, we shook the root on a platform (15 min, 110 rpm) to remove the soil from the root. The dislodged soil particles from the plant roots were considered rhizosphere soil. Soil 30 cm away from the root system was described as bulk soil. Then, the root, stem, and leaf were collected and washed with: (i) sterile Millipore water (30 s), (ii) 70% (v/v) ethanol (2 min), (iii) sodium hypochlorite solution (2.5% active Cl − with 0.1% Tween 80) (5 min), and (iv) 70% (v/v) ethanol (30 s). Finally, the samples were rinsed five times using sterile Millipore water. A sterile scalpel was used to separate the samples into small fragments and then soaked in a sterile phosphate saline buffer (PBS; 130 mM NaCl, 7 mM Na2HPO4, 3 mM NaH_2_PO_4_, pH 7.4) by employing a Polytron PR1200 mixer (Kinematica A6). Later, sterilization and homogenization of the samples were conducted under aseptic conditions in laminar airflow. Lastly, quadruplicate aliquots of each sample (1.5 ml) of the homogenized plant material were stored at −80°C awaiting DNA extraction.

### Measurement of sugarcane agronomic traits

The measurement of cane agronomic traits, namely, sugarcane heights, sucrose content, and stalk weight (kg stalk^−1^), followed by the theoretical production and yield parameters, was conducted in November 2019 using the method we adopted in our previous studies (Fallah et al., 2021; Pang et al., 2022).

### Assessment of soil nutrient

We investigated soil Si content by adopting the method used by Babu et al. (2016) study. In summary, an exact amount of filtrate was placed into a plastic centrifuge tube. Later, 10 ml of ddH_2_O and 0.5 ml of 1:1 hydrochloric acid (HCl) were added to the solution, followed by 1 ml of 10% ammonium molybdate solution (pH 7.0). Then, 1 ml of 20% tartaric acid solution was added, followed by 1 ml of the reducing agent amino naphthol n-sulphonic acid (ANSA). The solution was later mixed thoroughly at 2-min intervals after 5 min. UV–Vis spectrophotometer (Hach DR 5000) was used to estimate absorbance at 630 nm after 5 min. Finally, a UV–Vis spectrophotometer was concurrently used to assess Si content standard series absorbance readings (0, 0.2, 0.4, 0.8, 1.2, 1.6, and 2.0 mg L^−1^) organized in the same matrix. Soil physicochemical properties such as organic matter (OM), available potassium (AK), and available nitrogen (AN) were evaluated using methods leveraged by Fu et al. (2015) and Kwon et al. (2009), respectively. Whereas available phosphorus (AP) was assessed by adopting the approach employed by Pansu and Gautheyrou (2006), while soil pH was calculated using the method we leveraged in our previous studies (Bi et al., 2010).

### DNA extraction, PCR amplification, and illumina sequencing

We extracted total DNA from 500 mg of each sample (bulk soil, rhizosphere soil, root, stem, and leaf) using the PowerSoil DNA Isolation kit (Mo Bio Laboratories, Carlsbad, CA, United States) and Invisorb Spin Plant Mini Kit (Stratec Biomedical AG, Birkenfeld, Germany) following the manufacturer’s instructions. The quantity and quality of the DNA were assessed using the ratios of 260 nm/280 nm and 260 nm/230 nm. The DNA was later stored at −80°C until further processing.

The bacterial 16S rRNA gene V3–V4 region was amplified using a primer pair (Forward primer, 5′-ACTCCTACGGGAGGCAGCA-3′; reverse primer, 5′-GGACTACHVGGGTWTCTAAT-3′) combined with adapter sequences and barcode sequences. PCR amplification was performed in a total volume of 50 μl, which consisted 10 μl Buffer, 0.2 μl Q5 High-Fidelity DNA Polymerase, 10 μl High GC Enhancer, 1 μl dNTP, 10 μM of each primer, and 60 ng genome DNA. Below were the thermal cycling conditions: initial denaturation at 95°C for 5 min, followed by 15 cycles at 95°C for 1 min, 50°C for 1 min, and 72°C for 1 min, with a final extension at 72°C for 7 min. The PCR products obtained from the initial step of the PCR were purified using VAHTSTM DNA Clean Beads. The second round of PCR was then conducted in a 40 μl reaction, which consisted of 20 μl 2 × Phusion HF MM, 8 μl ddH_2_O, 10 μM of each primer, and 10 μl PCR products obtained from the initial step. Thermal cycling conditions were as follows: an initial denaturation at 98°C for 30 s, followed by 10 cycles at 98°C for 10 s, 65°C for 30 s and 72°C for 30 s, with a final extension at 72°C for 5 min. Lastly, Quant-iT™ dsDNA HS Reagent was employed to quantify all PCR products and pooled together. Illumina Hiseq 2500 platform (2 × 250 paired ends) at Biomarker Technologies Corporation, Beijing, China, was employed to conduct a high-throughput sequencing analysis of bacterial rRNA genes on the sample. Finally, the raw data were submitted to the NCBI Sequence Read Archive (accession no. PRJNA848385).

FLASH was adopted to conduct merge paired-end reads of the DNA fragments, using a sample-specific barcode appropriated to each sample. The sequences were then clustered at the same operational taxonomic unit (OTU) using 97% similarity. Later, sequences were accordingly chosen for each OTU to conduct the annotation of the taxonomic information for each sequence by employing the Ribosomal Database Project (RDP) (Cole et al., 2009). We also removed sequences with low quality if they did not correspond to the primer and barcode or if they did not exceed 200 nucleotides consisting of a high average quality score (*Q* ≥ 20) or no ambiguous base pairs. Sequences were then clustered at 97% nucleotide similarity. Finally, SILVA database (SILVA Release 138, Bacterial) was leveraged for the taxonomic classification of the bacteria respective sequences (Quast et al., 2013).

### Preparation of metabolic samples

Sugarcane tissue samples were extracted by adopting the method used by Chenkun et al. (2021) and Chen et al. (2013). In summary, the samples were ground into powder for 1.5 min at 30 Hz using a mixer mill (MM400, Retsch) after they were freeze-dried. The powder was later weighed (100 mg), followed by extraction during the night at 4°C with 0.8 ml 70% aqueous methanol (methanol: H_2_O_2_, 70:30, v/v) and pure methanol, followed by 10 min centrifugation at 10,000*g*. Supernatants were collected separately and mixed, and later filtrated (SCAA-104, 0.22 mm pore size; ANPEL Shanghai, China, www.anpel.com.cn/). Samples were mixed into five different tissue samples, namely leaf, stem, root, bulk, and rhizosphere soils to investigate the inter-tissue alterations in metabolites using nontargeted metabolomics analysis. Finally, we correspondingly mixed the samples into various quality control to conduct instrument stability.

### LC–MS/MS analysis

UHPLC system (1290, Agilent Technologies) with a UPLC BEH Amide column (1.7 μm 2.1 mm × 100 mm, Waters) combined with TripleTOF 5600 (Q-TOF, AB Sciex) was used to conduct LC–MS/MS analyses. The mobile phase comprised of 25 mM NH4OAc and 25 mM NH4OH in water (pH = 9.75) (A) and acetonitrile (B) was conducted using the following elution gradient: 0 min, 95% B; 7 min, 65% B; 9 min, 40% B; 9.1 min, 95% B; 12 min, 95% B, which was conveyed at 0.5 ml min^−1^, with an injection volume was 3 μl. To acquire MS/MS spectra on an information-dependent basis (IDA) during an LC/MS experiment, Triple TOF mass spectrometer was adopted.

### Data preprocessing and annotation

We converted the MS raw data (.d) files to the mzXML by employing ProteoWizard, and later processed it using R package XCMS (version 3.2). Later, we generated a data matrix that contained the mass-to-charge ratio (*m*/*z*) values, retention time (RT), and peak intensity. After XCMS data processing, R package CAMERA was used to conduct peak annotation. Finally, metabolites identification was conducted by employing an in-house MS2 database.

### Data analysis

We used Quantitative Insight into Microbial Ecology (QIIME2 v.1.9.1) (Caporaso et al., 2010) and R software (version 3.6.1) (Team, 2014) to examine bacterial community diversity (Shannon) (Keylock, 2005) and richness (ACE) (Chao and Lee, 1992). Bacterial community composition unique and overlap genera were visualized by constructing Venn diagrams (http://bioinfogp.cnb.csic.es/tools/venny/index.html). Principle coordinate analysis (PCoA) with Bray-Curtis distance was used to explore and visualize bacterial composition similarities or dissimilarities in the different plant tissues under both treatments. Source-tracking analysis was leveraged to identify the potential sources of observed bacterial communities in each host niche using Source Model of Plant Microbiome (SMPM) (Xiong et al., 2020). We further employed ADONIS analysis to have an in-depth understanding of how these bacterial community compositions in each plant tissue were altered under both treatments. To test the expression patterns of the abundant bacteria, both Bioconductor (http://www.bioconductor.org/) package ‘Mfuzz’ and R software (http://www.r-project.org/) based on fuzzy c-means were employed. Then, the fuzzification parameter was adjusted to *m* = 2 and the number of clusters to *c* = 12 to retain the soft clustering of all bacteria. PICRUSt2 was conducted to test soil bacterial metabolic function profile (KEGG) (Langille et al., 2013). Later, STAMP differential analysis was used to evaluate KEGG by comparing two plant tissues under the same treatment (Parks et al., 2014). Co-occurrence networks were further constructed to investigate the associations among soil bacteria, metabolite, and soil properties by following the descriptions in Zhang et al. (2021a) and Chen et al. (2020) studies. The connections between the abundant bacteria and metabolites in the network were visualized by leveraging a correlation matrix. Then, all potential pairwise Spearman’s rank was computed by employing Cytoscape version 3.6.1 (Shannon et al., 2003). A correlation among the abundant bacteria, metabolites, and soil properties was deemed statistically significant if Spearman’s correlation coefficient (*p*) was greater than 0.6 and the *p*-value was less than 0.05 (Junker and Schreiber, 2011). Lastly, the test data were calculated using ANOVA, which were then displayed by DPS software (version 7.05, www.dpssoftware.co.uk), and the differences between mean values of each treatment, plant, and soil compartment were compared using Tukey’s HSD test (*p* < 0.05).

## Results

### Assessment of sugarcane agronomic traits under Si utilization

Compared with CK, Si overwhelmingly promoted (*p* < 0.05) cane production and height, but revealed no significant difference for the rest of the parameters assessed. Although the sugarcane sucrose content did not increase significantly under the Si fertilizer treatment, it increased by 0.73 percentage points (Table 1).

**Table 1:** Effect of Si fertilizer on sugarcane agronomic traits.

	CK	Si
Stalk height (cm)	252.16 ± 3.61 b	**275.44 ± 4.50 a**
Stalk diameter (cm)	3.08 ± 0.05 a	3.14 ± 0.09 a
Sucrose content (%)	12.85 ± 0.29 a	13.58 ± 0.26 a
Available stalk number (hm^2^)	44,557 ± 1896 a	50,130 ± 1,256 a
Production (kg/hm^2^)	73523.6 ± 1703.9 b	**95612.6 ± 5227.6 a**
Single stalk weight (kg)	1.66 ± 0.10 a	1.91 ± 0.12 a

Sugarcane agronomic traits response to silicon fertilizer (Si) and control (CK). Different lowercase letters indicate a significant difference at *p* < 0.05. Values in bold means statistical significant.

### Response of soil physiochemical properties to Si amendment

Compared to CK treatment, soil AN marked a little difference in the rhizosphere soil compared with CK. We also noticed that soil AP in the rhizosphere soil peaked considerably (*p* < 0.05) under Si treatment than CK. Furthermore, soil AK in both the bulk and rhizosphere soils significantly increased (*p* < 0.05), whereas Si in the bulk soil significantly increased (*p* < 0.05) under Si treatment relative to that under CK. On the contrary, Si had no considerable impact on soil pH and soil OM compared with CK treatment (Table 2). MANOVA analysis further revealed that Si had a significant impact on soil AK and Si content, whereas different soil regions induced a significant change in soil AP, followed by soil AN and soil Si, while the interaction of CK and the various soil regions had a significant effect on soil AP, Si, and AK (Table 2).

**Table 2:** Effect of Si fertilizer on physical and chemical properties of sugarcane rhizosphere soil and bulk soil.

	AN	AP (mg/kg)	AK (mg/kg)	pH	OM (g/kg)	Si (mg/kg)
CK_Bulk	37.27 ± 6.67 ab	73.73 ± 6.07 c	42.00 ± 3.06 b	4.28 ± 0.08 a	23.32 ± 0.39 a	182.10 ± 3.53 b
Si_Bulk	21.53 ± 6.69 b	92.87 ± 3.82 bc	**154.33 ± 16.25 a**	4.25 ± 0.06 a	24.00 ± 0.86 a	**231.30 ± 5.16 a**
CK_Rhi	32.94 ± 10.38 ab	152.13 ± 16.96 b	38.67 ± 8.82 b	4.19 ± 0.12 a	24.53 ± 0.33 a	154.10 ± 2.57 c
Si_Rhi	52.38 ± 9.55 a	**312.67 ± 40.07 a**	**157.33 ± 44.76 a**	4.21 ± 0.04 a	23.62 ± 0.20 a	192.30 ± 2.77 b
Treatment (T)	NS	NS	***	NS	NS	**
Soil region (Sr)	*	**	NS	NS	NS	*
T*Sr	NS	***	**	NS	NS	***

***p < 0.001, **p < 0.01 and *p < 0.05. Values in bold means statistical significant.

### Bacterial community diversity

**Figure 1:**
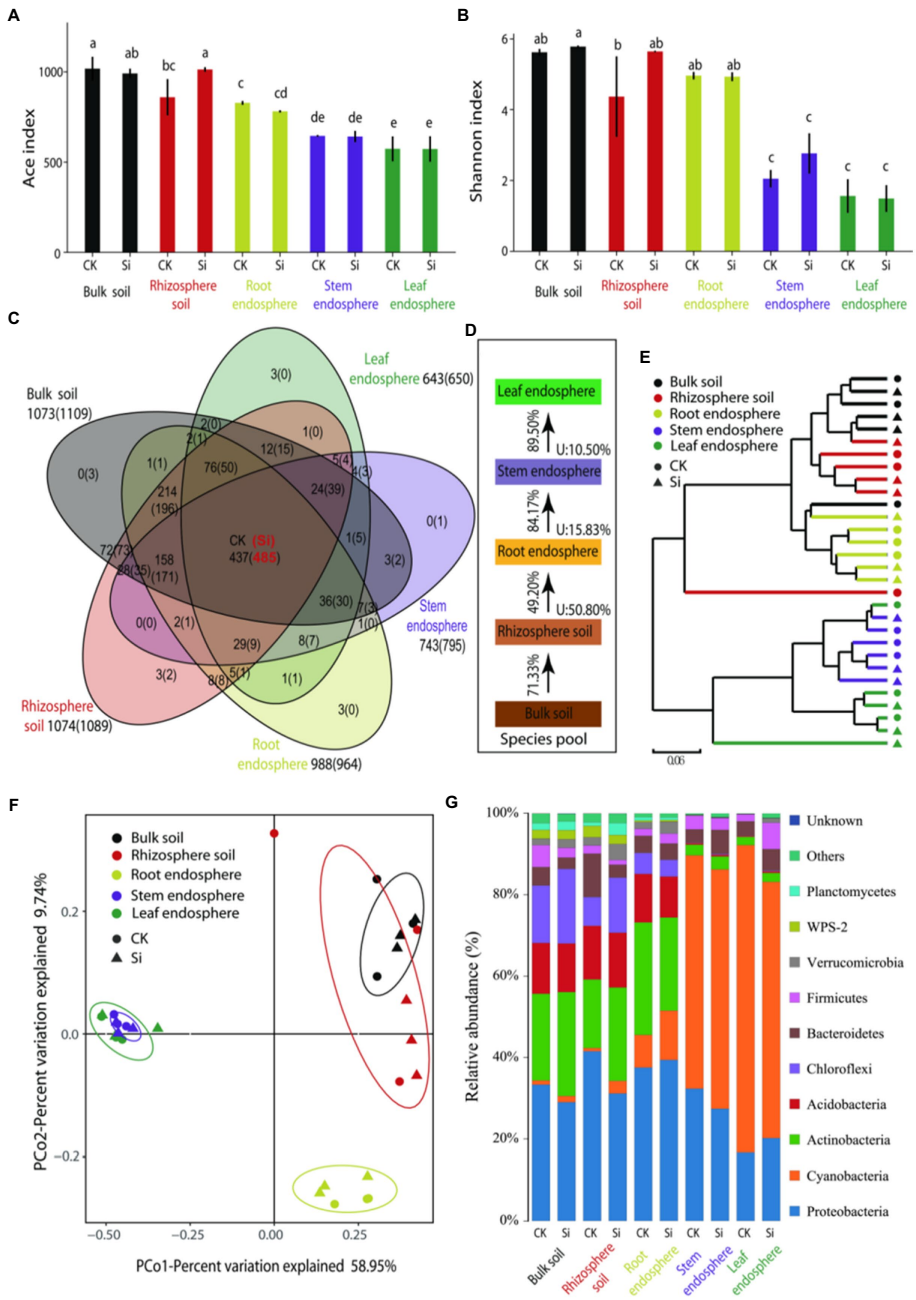
ACE index: bacterial richness index **(A)**, Shannon index: bacterial community diversity index **(B)**, a Venn diagram illuminating overlap and unique enriched bacteria OTU in the various plant and soil compartments **(C)**, Source Model of Plant Microbiome (SMPM) illustrating the potential sources of bacterial community composition in the different plant and soil compartments under both treatments, “U” denotes the proportion of bacterial community composition from unknown source **(D)**. Followed by hierarchical clustered heatmap of all the bacteria in the different soil and plant compartments under both treatments **(E)**, principal coordinate analysis (PCoA) with Bray-Curtis distance showing similarities and dissimilarities of bacterial phyla under the both Si and CK and various soil and plant compartments **(F)**, and the relative abundance of bacterial community composition in the entire sample **(G)**. Different lowercase letters signify significant differences between various compartment under both treatments based on the LSD test (*p* < 0.05).

With the adoption of high-throughput Illumina sequencing, a total of 2,399,532 raw reads were generated, and a total of 2,241,717 were obtained after quality assessment. The reads were clustered into 1,147 OTUs at a 97% sequence similarity level. The remaining numbers of reads ranged from 49,250 to 73,750 among samples, and we rarefied each sample to the minimum size (49,250) (Supplementary Table S1). The rarefaction curves of OTU richness and Shannon diversity per compartment reached a saturation plateau, thus suggesting that we have sampled most of the diversity in the soil and sugarcane microbiome. The shape of the curves demonstrated that the OTU richness (ACE) and diversity (Shannon) were consistently high in the rhizosphere, followed by the root endosphere (Supplementary Figure S1). We also examined bacterial ACE and the Shannon in each sample (Figures 1A,B). There were obvious differences in bacterial diversity and richness in different locations. OTU richness was highly dependent on plant compartment (*p* < 0.05), consisting of high richness values of 1017.7 ± 66.0 (bulk soil) and 859.4 ± 100.5 (rhizosphere soil), while bacterial richness in the root samples (828.1 ± 11.0) and stem samples (645.4 ± 5.0) consistently decreased. OTU richness indices of the leaf samples (573.8 ± 68.8) were comparable with the stem samples. In the rhizosphere, OTU richness was more pronounced in the Si treatment compared with CK treatment (Figure 1A). For bacterial diversity, we also observed an obvious separation between the soil samples and endosphere samples (*p* < 0.05). Higher diversity was observed in the bulk soil, rhizosphere soil, and root endosphere as compared to the samples of the stem and leaf tissues (Figure 1B).

The Venn diagram results further showed that the number of OTUs was significantly high (1073), but decreased considerably in the leaf endosphere (643), of which 437 OTUs were present in other compartments (Figure 1C). This suggests that most of the bacteria may have migrated from the soil to the different sugarcane compartments. However, except for the root endosphere, the number of bacterial OTUs in the different compartments increased by different degrees under Si treatment. There were 485 OTUs in the different plant and soil compartments. This further showed that compared with the CK treatment, Si can improve the diversity and connectivity of bacteria in different parts (Figure 1C).

Source-tracking analysis was conducted to identify the potential sources of observed bacterial communities in each host niche. The Source Model of Plant Microbiome (SMPM) suggested that crop-associated bacterial communities were mainly migrated from the bulk soils and gradually filtered in different plant compartment niches. Only 49.2% of the bacteria in the rhizosphere soil entered the roots, indicating that the bacteria were strongly selected at the soil–plant interface (Figure 1D). The root endosphere, stem endosphere, and leaf endosphere potentially accommodated the majority of taxa from a nearby species pool, with the known source value of approximately 84.17%, exceeding that in the soil–plant interface. This distribution pattern was further validated by cluster analysis, where bacteria in the aboveground tissues were distinctly separated from the belowground (Figure 1E).

We evaluated beta diversity at the OTU level (OTUs defined at a 97% similarity cut-off). Principle coordination analysis (PCoA) reinforced the distribution trend observed in the bacterial community structures dissimilarities among the various compartments. The results further showed that PCo1 explained 58.95% and PCo2 9.74% of the total variation. The subsurface bacteria (bulk/rhizosphere soil and root endosphere) and the aboveground bacteria (stem and leaf endosphere) were separated on the first main axis. We also observed that Si treatment had little impact on the bacterial community than in the different compartments (Figure 1F).

### Abundant bacterial in each compartment

It can be seen from Figure 1G that there were obvious differences in the composition of the bacterial phyla in the different locations. The belowground bacteria were mainly composed of *Proteobacteria*, *Actinobacteria, Acidobacteria,* and *Chloroflexi*, accounting for 80.72% of the total abundance, while the aboveground bacteria were mainly composed of *Cyanobacteria* and *Proteobacteria*, representing 90.94%. Furthermore, we used analysis of variance (LSD) to evaluate the effect of Si and the diffident compartments on bacteria whose relative abundance was greater than 1% (Supplementary Table S4). Virtually all identified bacterial phyla displayed a significant compartment effect, except for *Bacteroidetes*. In the bulk soil, rhizosphere soil, and roots endosphere samples, we observed a significant enrichment (*p* < 0.05) of *Actinobacteria* (relative abundance = 21.3%, 16.9%, 27.7%), *Acidobacteria* (relative abundance = 12.5%, 13.2%, 11.9%), and *Chloroflexi* (relative abundance = 14.1%, 7.0%, 5.2%), while *Cyanobacteria* were significantly depleted (*p* < 0.05) as compared to the stems and leaves compartments. The abundance of *Proteobacteria* was significantly lower in the cane leaf compared to the other compartments. In the bulk soil and rhizosphere soil samples, WPS-2 relative abundance accounted for 2.35%, followed by *Planctomycetes* (1.90%) as compared to the endosphere compartments. For the genus level, we found a total of 381 genera (Supplementary Figure S2; Supplementary Table S2; Supplementary Sheet 1), we defined the core bacterial as the 20 most abundant genus in each of the compartments, thus resulting in 71 genera (Supplementary Table S2; Supplementary Sheet 2). The percentages of the total community covered by the core genus ranged from 72.9% (bulk soil) to 77.4% (rhizosphere soil), and from 76.1% (root) to 71.7% (stem), and 72.0% (leaf). We observed significant compartment effects across all core bacterial genera. In the bulk soil, genera such as c_AD3 (5.54%), 7,703 (1.21%), *Lactobacillus* (0.78%), and *Romboutsia* (0.38%) were more pronounced (*p* < 0.05) as compared to the rhizosphere soil, root, stem and leaf compartments. In the root samples, *Pajaroellobacter* (3.30%), *Salix_integra* (1.30%), and *Thermosporothrix* (1.83%) marked significant enrichment (*p* < 0.05) as compared to the other compartments. We also notice that *Aegilops-tauschii* and *Triticum-aestivum-bread-wheat* peaked considerably in stem and leaf samples compared with the soil and root samples. However, *Conexibacter, Acidibacter*, *Acidothermus*, *Singulisphaera, Bradyrhizobium, Burkholderia-Caballeronia-Paraburkholderia, Catenulispora, Mycobacterium, Roseiarcus, Actinospica,* and *Dyella* were significantly (*p* < 0.05) depleted in the stem and leaf samples. In addition, it was noticed that Si had significant effects on all the core bacterial genera, particularly in the rhizosphere, bulk soil, root, and stem endosphere.

To statistically support the visual clustering of the bacterial communities in the PCoA analysis (Figure 1F), different plant and soil compartments and Si treatment were examined using ADONIS method (Table 3). Under Si treatment, no significant difference in bacterial composition between bulk soil and rhizosphere soil was observed, but a difference in bacterial function (*p* = 0.046) was observed. The result revealed that all the different compartments rendered bacteria significantly dissimilar from each other at the phylum, genus, and functional levels. Compared with the control, there was no difference observed in bacterial composition and function in the bulk soil and sugarcane leaves under Si. However, there were differences in bacterial composition and function in the rhizosphere soil (*p* < 0.05). On the other hand, the function of endophytic bacteria (genus level) in the roots and stems changed significantly under Si treatment (Table 3).

**Table 3:** Adonis showed the difference in bacterial composition in two treatments and different compartments.

ADONIS output	Phylum		Genus		Functional	
	*R*	*P*	*R*	*P*	*R*	*P*
Bulk soil vs. rhizosphere soil	0.143	0.343	0.157	0.307	0.223	0.046
Bulk soil vs. root	0.5	0.02	0.533	0.016	0.446	0.031
Bulk soil vs. stem	0.92	<0.001	0.765	0.003	0.97	0
Bulk soil vs. leaf	0.939	<0.001	0.561	0.013	0.935	<0.001
Rhizosphere soil vs. root	0.502	0.02	0.263	0.133	0.312	0.09
Rhizosphere soil vs. stem	0.654	0.006	0.508	0.019	0.877	<0.001
Rhizosphere soil vs. leaf	0.76	0.003	0.376	0.054	0.83	0.001
Root vs. stem	0.928	<0.001	0.821	0.002	0.979	0
Root vs. leaf	0.946	<0.001	0.583	0.011	0.945	0.001
Stem vs. leaf	0.592	0.01	0.216	0.192	0.485	0.023
CK vs. Si (bulk soil)	0.229	0.098	0.206	0.061	0.169	0.178
CK vs. Si (rhizosphere soil)	0.217	0.041	0.215	0.012	0.228	0.033
CK vs. Si (root)	0.088	0.358	0.199	0.064	0.335	0.031
CK vs. Si (stem)	0.227	0.127	0.233	0.039	0.233	0.073
CK vs. Si (leaf)	0.018	0.725	0.057	0.599	0.026	0.681

### Expression patterns of bacterial abundance in compartments

**Figure 2:**
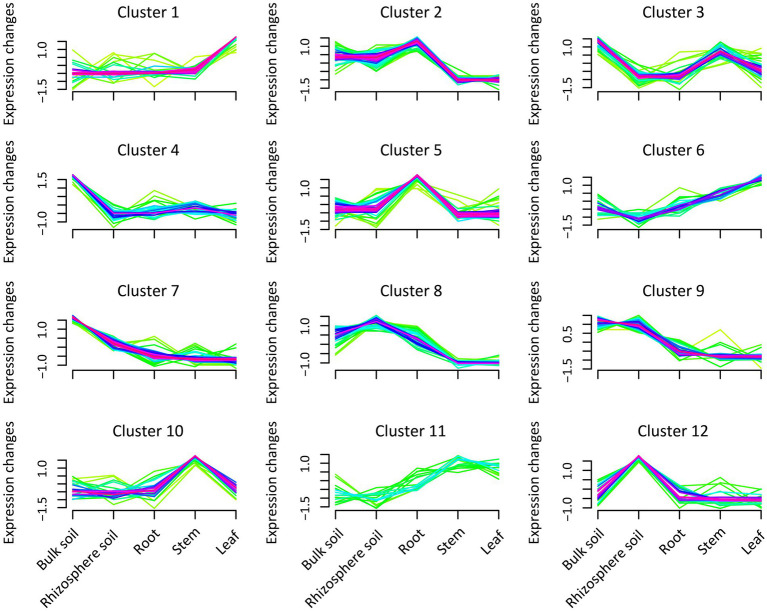
Expression pattern of bacteria abundance in the different plant and soil compartments (bulk and rhizosphere soils, leaf, stem, and root). Green indicates low abundance values, and purple represents the cluster expression trend of the abundant bacteria.

We conducted a cluster analysis to interpret the expression patterns of bacterial abundance in different plant and soil compartments (Figure 2; Supplementary Table S5). The analysis demonstrated that the expression patterns of bacterial communities could be divided into 12 categories. In clusters 4 and 7, the abundance of bacteria peaked significantly in the bulk soil compared with the other compartments, mainly occupied by *Lactobacillus*, *Alcaligenes*, *Bacillus*, *Streptococcus,* and *Nitrospira*. In cluster 12, it was also revealed that the abundance of bacteria in rhizosphere soil was higher than that in the other compartments, particularly driven by *Gemmatimonas*, *Flavisolibacter,* and *Streptomyces*. Furthermore, *Blastococcus*, *Rhizomicrobium,* and *Aquisphaera* expression patterns peaked in cluster 9 in the rhizosphere and bulk soils. In clusters 2 and 8, the analysis also showed that the abundance of bacteria in the soil and roots was higher than that in the stems and leaves, potentially driven by *Sphingomonas*, *Mesorhizobium,* and *Burkholderia-Caballeria-Paraburkholderia*. While *Cetobacterium*, *Amnibacterium*, *Akkermansia,* and *Ruminococcus_UCG-002* abundance in sugarcane stems were more pronounced in cluster 10 than that in the other compartments. Whereas clusters 1 and 6 were predominantly driven by *Lysobacter, Caulobacter,* and *Allorhizobium-Neorhizobium-Pararhizobium-rhizobium* in leaves tissue compared with the other compartments.

### Ternary mapping analysis reveals the composition of enriched or depleted bacterial communities in sugarcane and soil compartments

**Figure 3:**
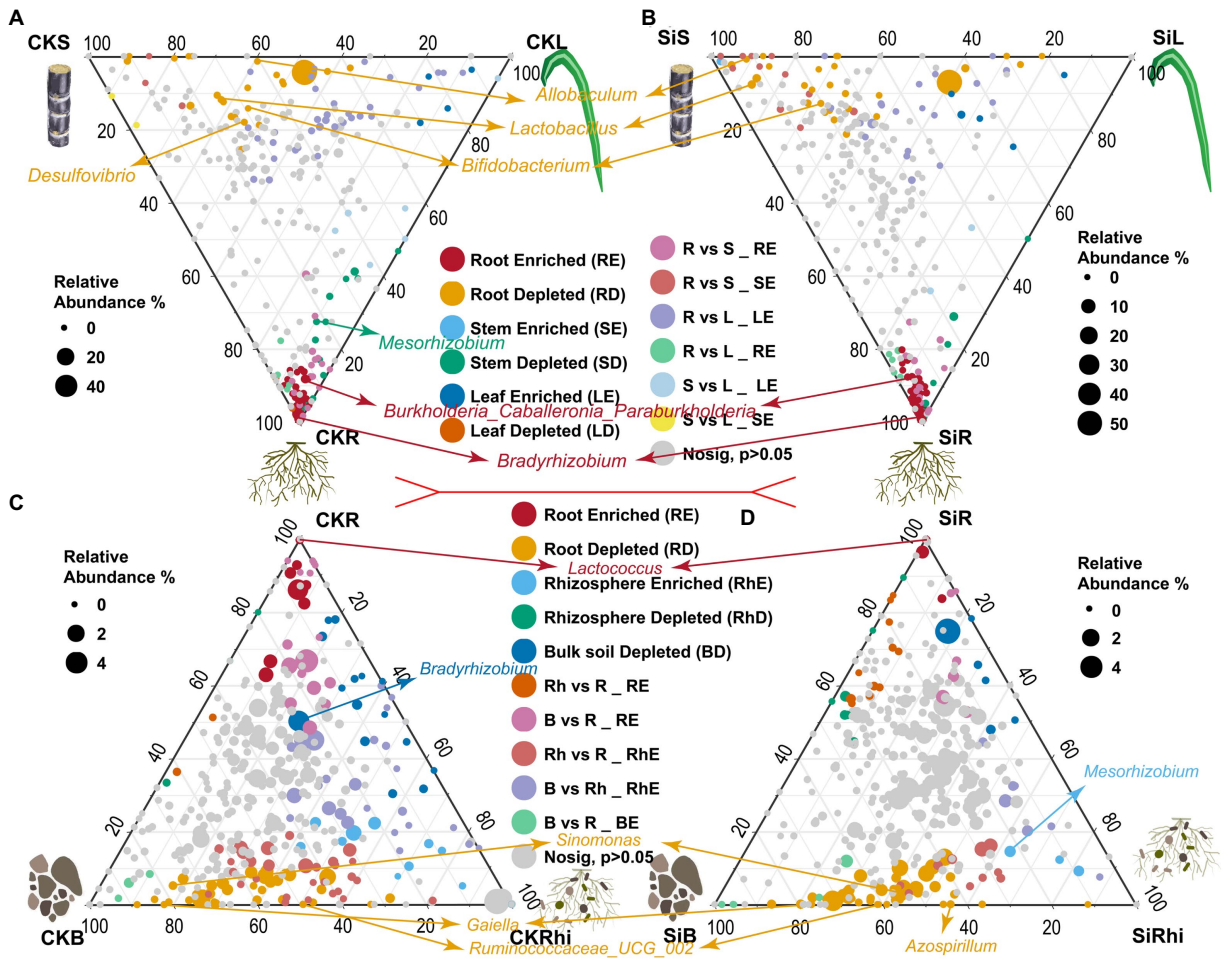
Ternary plot illustrating depleted and enriched bacterial community composition in the different plant and soil compartments. Each point represents the depleted or enriched bacterial community composition. The location of each point symbolizes the relative abundance of bacteria in each region, and its size denotes the average across three compared regions. Each colored circles represent depleted or enriched bacterial community composition in one region relative to the other: CKS, sugarcane stem in CK treatment; CKL, sugarcane leaf in CK treatment; CKR, sugarcane root in CK treatment; CKB, bulk soil in CK treatment, and CKRhi, sugarcane rhizosphere soil in CK treatment. Followed by SiS, sugarcane stem in silicon amended soil; SiL, sugarcane leaf in silicon amended soil; SiR, sugarcane root in silicon amended soil; SiB, bulk soil in silicon amended soil, and SiRhi sugarcane rhizosphere soil in silicon amended soil.

DESeq2 was used to compare the differences of bacterial species in the stem, leaf, and root under CK (CKS *VS* CKL *VS* CKR), and Si treatment (SiS *VS* SiL *VS* SiR) to investigate the enriched or depleted bacteria (Figures 3A–D; Supplementary Table S6). It was observed that the enrichment of endophytic bacteria in different sugarcane compartments was different. For instance, *Allobaculum, Lactobacillus,* and *Bifidobacterium* were significantly depleted in the stem, while *Burkholderia, Caballeronia, Paraburkholderia,* and *Bradyrhizobium* in sugarcane root were significantly higher than that in sugarcane stem and leaf under both CK and Si. It was also noticed that *Desulfovibrio* was significantly enriched only in the stem of the CK, while *Mesorhizobium* revealed the opposite (Figures 3A,B). Moreover, bacterial abundance in the root, rhizosphere soil, and bulk soil exhibited distinct patterns. In the CK and SI treatments, *Lactococcus* in root was significantly higher than that in soil. However, *Sinomonas*, *Gaiella,* and *Ruminococcaceae UCG* abundance significantly diminished relative to those in the soil compartment (Figures 3C,D). In addition, *Bradyrhizobium* in the bulk soil of the CK was significantly lower than that in the rhizosphere soil and roots (Figure 3C). Whereas *Mesorhizobium* was significantly higher in the rhizosphere soil than that in the bulk soil and root. However, *Azospirillum* in the root demonstrated the opposite compared with the bulk soil of Si treatment (Figure 3D).

### Changes of bacteria in various parts under Si treatment

**Figure 4:**
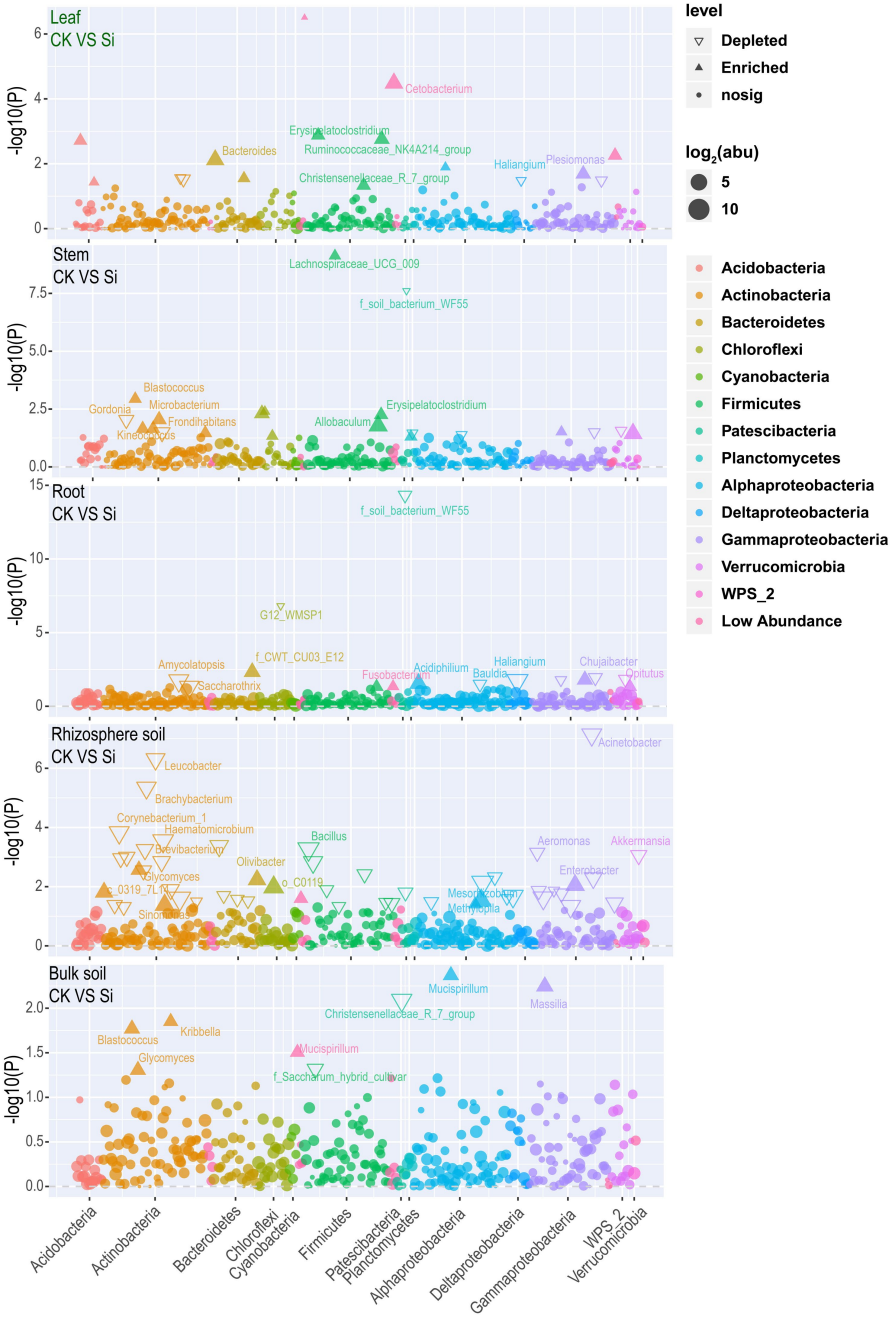
Manhattan plot showing bacterial community composition in each plant and soil compartment under Si compared with CK. Each triangle or dot signifies a single genus. Genus enriched in CK or Si are represented by filled or empty triangles, respectively (FDR adjusted *p* < 0.05, Wilcoxon rank-sum test). Genus are arranged in taxonomic order and colored according to the phylum, while Proteobacteria was assessed at the class level to have a better insight into their classification.

Later, the result was visualized using a Manhattan plot to have a comprehensive insight into the effect of Si on bacteria abundance in each of the various plant and soil regions. The results showed that Si significantly improved *Cetobacterium, Bacteroides, Erysipelatoclostridium, Plesiomonas, Ruminococcaceae_NK4A214_group*, *Brevinema, Christensenellaceae_R_7_group, Edaphobacter,* and *Aliidongia* in the leaf tissue compared with CK. In contrast, *Labedaea, Dokdonella, Crossiella,* and *Haliangium* were significantly reduced. It was noticed that Si significantly increased *Lachnospiraceae_UCG_009*, *Blastococcus, Erysipelatoclostridium, Microbacterium, Allobaculum, Frondihabitans, Kineococcus, Ellin6067,* and *Akkermansia* in the stems. However, *Gordonia, Yonghaparkia, Aquisphaera,* and *Methylovirgula* revealed a decreasing trend. In the sugarcane root, Si significantly increased *Acidiphilium, Fusobacterium,* and *Allobaculum*. On the other hand, C*hujaibacter, Opitutus*, *Amycolatopsis, Haliangium, Bauldia,* and *Saccharothrix* significantly diminished. Furthermore, Si significantly increased the *Mesorhizobium, Sinomonas, Enterobacter, Olivibacter, Glycomyces, Mucispirillum,* and *Methylopila* in the rhizosphere. On the contrary, the analysis also revealed that Si significantly reduced *Ochrobactrum, Amycolatopsis, Bacillus, Haematomicrobium, Alcaligenes, Aquicella, Corynebacterium_1, Acinetobacter, Brachybacterium, Lactobacillus, Leucobacter, Pseudomonas, Aeromonas, Brevibacterium, Akkermansia,* and *Arthrobacter*. In Bulk soil, Si significantly improved *Blastococcus, Glycomyces, Kribbella, Massilia,* and *Mucispirillum,* but *Christensenellaceae R 7 group* and *Saccharum hybrid cultivar* decreased significantly (Figure 4; Supplementary Table S7).

### Variation of bacterial function in different soil and plant compartments under Si amended soil

**Figure 5:**
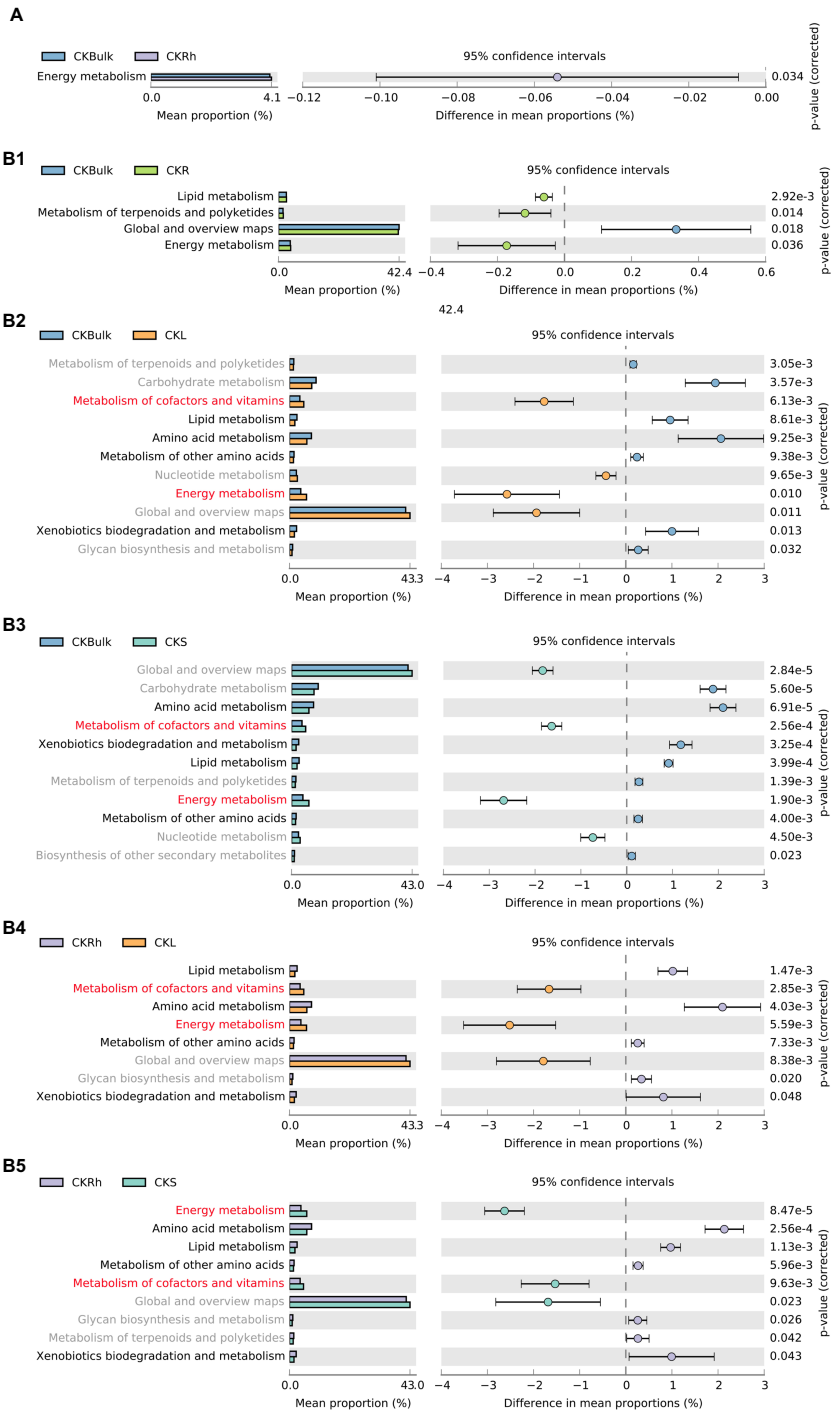
Extended error bar graphs specifying the significant difference in bacterial functional guilds at level 2 in each compartment under CK **(A)** (*p* < 0.05, average proportion, *n* = 3). The points illustrate differences between CKBulk, bulk soil in CK treatment and CKRh, sugarcane rhizosphere soil in CK treatment; CKBulk and CKR, sugarcane root in CK treatment; CKBulk and CKL, sugarcane leaf in CK treatment; CKBulk and CKS, sugarcane stem in CK treatment, followed by CKRh, and CKL, and CKRh and CKS, respectively.

In the different compartments, we assessed variations of bacterial function under the CK treatment. The analysis demonstrated that energy metabolism increased significantly in rhizosphere soil compared to the bulk soil (Figure 5A). By comparing the metabolic functions of endophytic bacteria in the different soil compartments, we also found that the metabolism of cofactors and vitamins, and energy metabolism were significantly enriched in the leaf tissue. We also noticed that amino acid metabolism, metabolism of other amino acids, xenobiotics biodegradation and metabolism, and lipid metabolism in bulk and rhizosphere soils were significantly increased (Figure 5B). We also observed significant differences in bacteria functions after bacteria in the stem and root were compared with those in compartments under CK (Supplementary Figure S3A). For instance, energy metabolism, global and overview maps, metabolism of cofactors and vitamins, and nuclear metabolism in stem and leaf tissues were significantly higher than those in the root. Furthermore, bacteria metabolic function in the rhizosphere soil and sugarcane root changed (Table 3) after Si amendment. We also observed a similar pattern after we compared the metabolic function of endophytic bacteria between the CK and Si treatments in sugarcane roots. For instance, nucleotide metabolism was significantly enriched in Si treatment, but amino acid metabolism and metabolism of other amino acids decreased significantly (Supplementary Figure S3B).

### Exploring the associations between bacterial community structure and different plant and soil compartments under Si amendment

**Figure 6:**
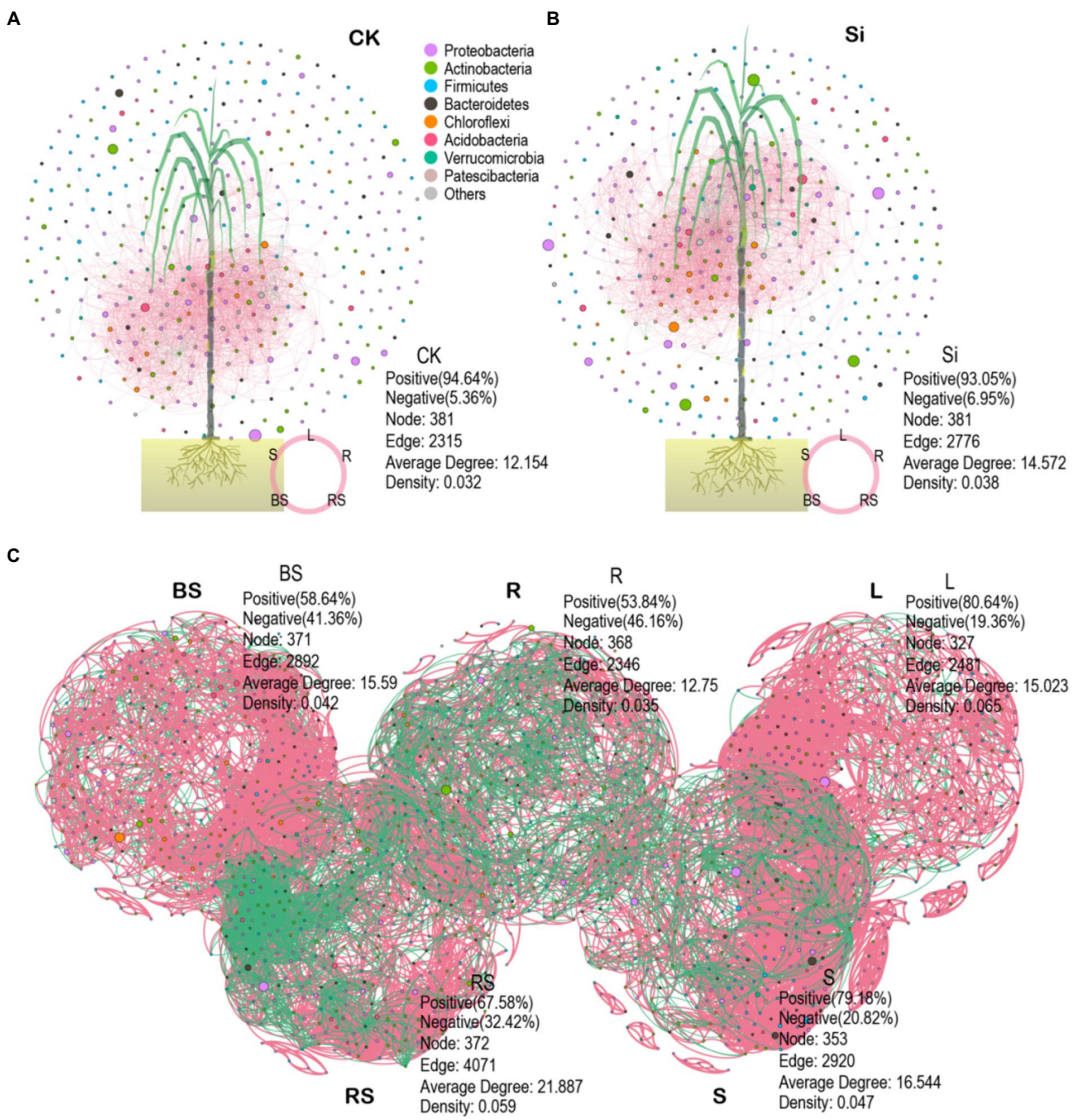
Co-occurrence networks depicting the entire bacteria in the different plant and soil compartments under CK **(A)**, Si amendment **(B)**, and bacterial community composition in each compartment (BS, bulk soil; R, root; L; leaves; RS, rhizosphere soil; S, stem) **(C)**. The data in the picture represent the topological role of the microbiome network and nodes. A node represents a genus. The green and red lines indicate negative and positive interaction, respectively.

We also explored the topological properties of the symbiotic network composed of microbial communities in the different plant and soil compartments (Table 4). It was observed that the total number of nodes and links, including the network of rhizosphere soil showed greater complexity compared with the other compartments. We further constructed an interactive network of plant–soil systems and bacteria to better visualize the relationship between the bacterial community in each treatment (Figures 6A,B), and in each compartment (Figure 6C). The analysis showed that the network topological parameters such as the number of network nodes, the number of connections, the aggregation coefficient, the characteristic path length, the network density, and the average connectivity were distinct between CK and Si. In the control group, the interaction network between soil and sugarcane bacteria revealed 381 nodes and 2,315 lines, accounting for 94.64% positive correlation (Figure 6A). In the Si treatment, the number of interaction network nodes of bacteria between the soil and sugarcane compartments revealed no difference compared with CK, but the number of connections increased to 2,776, representing 93.05% positive correlation. It was also revealed that the number of network connections and the positive connection of bacteria interaction network in (soil-sugarcane) system increased under Si amended soil. Compared with CK, the bacteria interaction network of the soil-sugarcane system showed higher network density, aggregation coefficient, and average connectivity under Si treatment (Figure 6B; Table 4). This suggests that Si amendment has the potential to increase plant–soil systems bacteria connections, thereby increasing the functional diversity in the host plant, which, in turn, had a positive growth effect on sugarcane growth. Co-occurrence network results of bacterial genera in the different compartments showed that the number of nodes and the number of significant connections between bacteria was more pronounced in the rhizosphere soil, indicating that the rhizosphere soil may be the most complex area for bacteria activities in the soil–plant system (Figure 6C; Table 4).

**Table 4:** Topological features of networks of the different treatments and soil–plant-associated bacterial communities in the various compartments based on Spearman’s correlation method.

	CK	Si	BS	RS	R	S	L
*R*-Value	>0.8		>0.85				
*p*-Value	<0.05						
N	15	15	6	6	6	6	6
Nodes	381	381	371	372	368	353	327
Edges	2,315	2,776	2,892	4,071	2,346	2,920	3,481
Positive (%)	94.64	93.05	58.64	67.58	53.84	79.18	80.64
Negative (%)	5.36	6.95	41.36	32.42	46.16	20.82	19.36
Average degree (avgK)	12.152	14.572	15.59	21.887	12.75	16.544	21.291
Diameter	7	7	8	8	8	7	7
Density	0.032	0.038	0.042	0.059	0.035	0.047	0.065
Average clustering coefficient (avgCC)	0.601	0.617	0.577	0.612	0.526	0.565	0.611
Average path distance (GD)	2.179	2.146	3.749	3.449	3.677	3.401	3.412

### Assessment of the associations among metabolites, bacterial community structure, and soil properties

**Figure 7:**
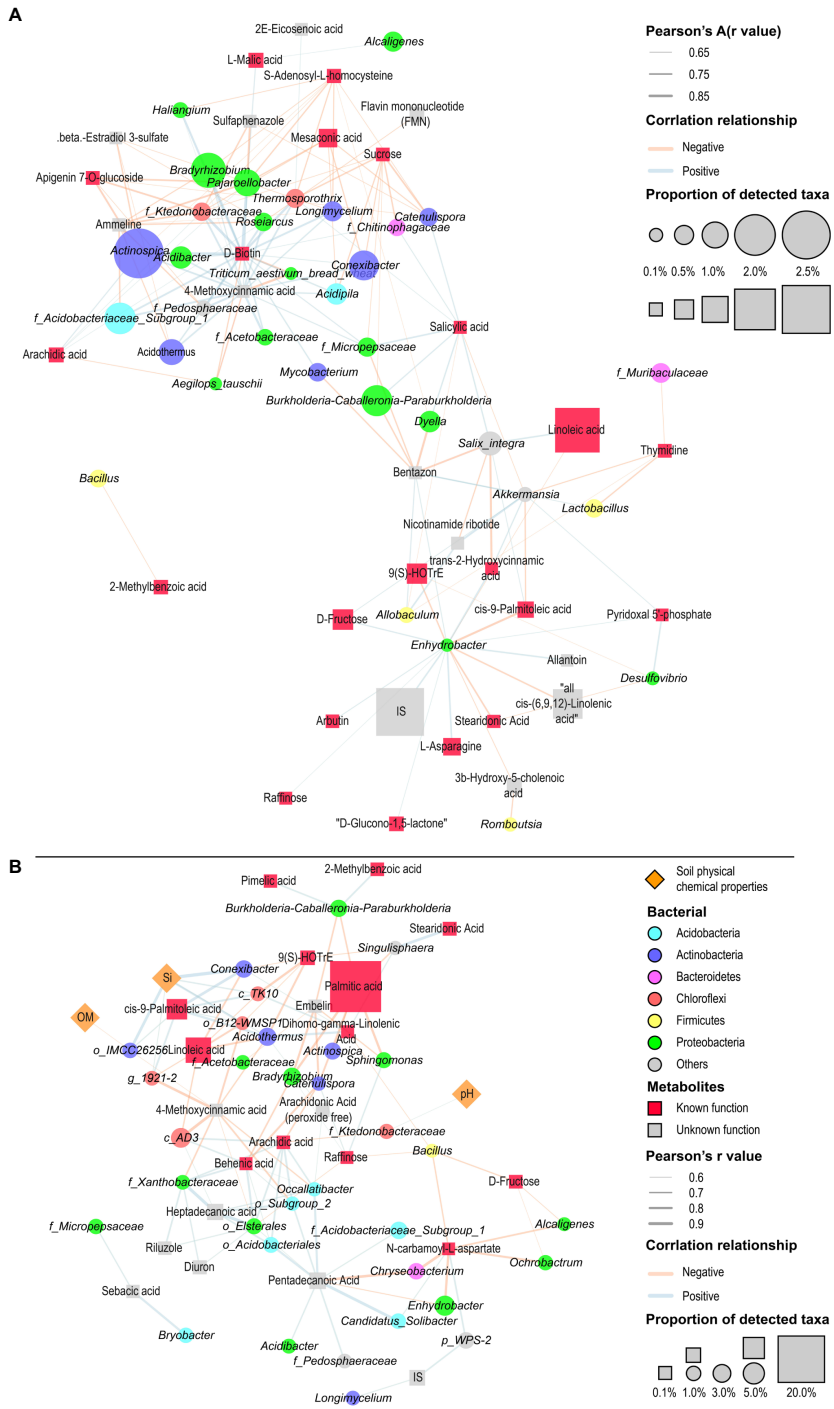
Correlation network illuminating the association between bacterial community composition and metabolites in the different sugarcane tissues (root, stem, and leaf) **(A)**, and among soil biochemical properties, metabolites, and bacterial community composition in the different soil regions (bulk and rhizosphere soils) **(B)**. Blue and red lines depict positive and negative correlations, respectively. Square boxes with orange and red color represent soil physiochemical properties and metabolites, respectively, while circles in different colors represent bacteria. Different colored circles represent different bacterial phyla. Different colored squares represent metabolites classified according to the known Human Metabolome Database. Different sizes of squares and circles indicate relative abundance. The thickness of the connecting line indicates the absolute value of the correlation *R*-value.

To establish potential associations of metabolites and bacteria in the various sugarcane and soil regions, we identified 51 core metabolites (Supplementary Table S3; Supplementary Sheet 2) from the top 20 high abundance metabolites in the various plant and soil regions using the entire sample of metabolome data (Supplementary Table S3; Supplementary Sheet 1). We analyzed the correlation between core metabolic substances and core bacterial composition (Supplementary Table S2; Supplementary Sheet 2) in the different plant tissues (root, stem, and leaf), and visualized them with Cytoscape software (Figure 7A). The results showed that the metabolites in the different plant tissues had distinct effects on bacterial community composition. In the various plant compartments, D-biotin showed a significant positive correlation with the *Pajaroellobacter, Actinospica, Thermosporothrix, Haliangium,* and *Bradyrhizobium*. Furthermore, d-fructose was positively correlated with *Enhydrobacter* and *Akkermansia*, while raffinose was positively correlated with *Enhydrobacter*. It was also noticed that salicylic acid (SA) was positively correlated with *F_micropipesaceae, Dyella, Salix Integra, Burkholderia Caballeria Paraburkholderia, Catenulispora, f_chitinophagaceae,* and *Thermosporothrix*. However, SA was negatively correlated with *Akkermansia* and *Allobaculum*. Our analysis also revealed that S-adenosyl-l-homocysteine was negatively correlated with *Pajaroellobacter, Thermosporothrix, Longimycelium, Bradyrhizobium, Haliangium, Catenulispora, Dyella, and Actinospica*. Additionally, sucrose showed a significant negative correlation with *Catenulispora, f_Chitinophagaceae, Thermosporothrix, Acidipila, Conexibacter, f_Ktedonobacteraceae, f_micropipesaceae,* and *Burkholderia-Caballeria*-*Paraburkholderia*. Sucrose also exhibited a significant negative correlation with *Acidipila, Burkholderia-Caballeronia-Paraburkholderia, Catenulispora, Conexibacter, Thermosporothrix* (Figure 7A; Supplementary Table S8).

Later, we elucidated the association among bacterial community, key metabolites and soil properties in the different soil regions (rhizosphere and bulk soils) (Figure 7B). It was shown that d-fructose, raffinose, dihomo-gamma-linolenic acid, and N-carbamoyl-l-aspartate were negatively correlated with *bacillus* abundance (*R* < −0.6). Moreover, there was a significant negative correlation between the rich palmitic acid, *Actinospica*, *Bradyrhizobium*, *Burkholderia-Caballeronia-Paraburkholderia*, *Catenulispora, Singulisphaera,* and *Sphingomonas*. Linoleic acid and CIS-9-Palmitolic acid were negatively and positively correlated with *Acidothermus* and *Conexibacter*, respectively. The analysis also revealed that arachidic acid was negatively correlated with *Catenulispora* and *f_Ktedonobacteraceae*. However, arachidic acid exhibited a positive correlation with *f_Xanthobacter* and *Occallatibacter*. Whereas dihomo-gamma-linolenic acid was positively correlated with *Acidothermus*, *Conexibacter,* and *Singulisphaera*. While raffinose exhibited a positive association with *Bradyrhizobium*, *Catenulispora* and *Sphingomonas*. Correlation network analysis also revealed that soil physicochemical properties had a significant association with metabolites community, but revealed a minimum relationship with the bacterial community. For example, the content of soil Si was positively correlated with the *Acidothermus*, *Conexibacter,* and *OM o_IMCC26256*. The result also showed that soil pH demonstrated a significant positive correlation with *f_Ktedonobacterae* (Figure 7B; Supplementary Table S8).

## Discussion

Our understanding of the impacts of Si application on soil physicochemical properties, microbial composition, metabolisms, and sugarcane productivity is limited. Therefore, our findings could broaden our understanding of how Si influences soil fertility and productivity, as well as microbial and metabolisms communities in different soil and sugarcane compartments. Here, we observed that the application of Si not only improved sugarcane agronomic parameters, but also promoted its yield, which is consistent with previous findings, where Si application enhanced the yield and quality of rice (Song et al., 2021) and sugarcane (Meyer and Keeping, 2000). It was also observed that Si amended soil remarkably enhanced soil nutrient status, especially Si, AP, and AK, which is in agreement with the finding documented by Zhang et al. (2021b) and Matychenkov and Ammosova (1996). We, therefore, concluded that supplementing the soil with Si could enhance soil nutrient status, which, in turn, are made available to the plant, evident by the significant increase in cane agronomic parameters.

A number of studies have documented that soil ecosystems and plant tissues, including root, stem, leaf, flower, and fruit are heavily colonized by different microbes (Beckers et al., 2017). The colonization of these microbes vary distinctly from one compartment to another (Tardif et al., 2016; Xiong et al., 2020). Likewise, we observed that OTUs richness was highly dependent on plant compartment, where OTUs richness consistently decreased in value from 1017.7 ± 66.0 (bulk soil), to 859.4 ± 100.5 (rhizosphere soil), followed by the root samples (828.1 ± 11.0), the stem samples (645.4 ± 5.0) and the leaf samples (573.8 ± 68.8). Similarly, bacterial diversity demonstrated tissue-dependent distribution patterns, with the bulk soil, rhizosphere soil, and root endosphere revealing the highest amount of bacterial diversity compared with the stem and leaf tissues. These findings have been previously suggested for many plants such as maize (Xiong et al., 2021), sugarcane (de Souza et al., 2016), and agaves (Coleman-Derr et al., 2015), wherein it was observed that variations in microbes diversity and richness were compartment-dependent. For bacterial diversity, we also observed a similar pattern, where bacterial diversity in the bulk soil, rhizosphere soil and root endosphere peaked significantly compared with the stem and leaf tissues under both treatments. Interestingly, these distinct variation patterns in the microbial community structure were further validated by PCoA, Adonis, and DESeq2 analyses. Thus suggesting that the distinct distribution patterns observed in the bacterial community structure were component-specific. We, therefore, assumed that the gradual decrease in the OTUs richness and diversity from belowground to aboveground suggests that these bacteria might have initially originated from the soil and moved to the aboveground components of the plant, as displayed by the Source Model of Plant Microbiome (SMPM) analysis. Another reason for this phenomenon could be largely ascribed to the host’s selection of bacteria, such as the expression of a series of genes related to the resistance of plant innate immune response (Jones and Dangl, 2006; Hardoim et al., 2008), as well as the exposure of the ground parts to harsh conditions, such as ultraviolet radiation and diurnal temperature fluctuations. These immune responses and environmental conditions may have eventually led to the decrease in the diversity index and richness index of endophytic bacteria in the leaf and stem tissues.

Although Si application exhibited no significant regulatory effect on the endophytic bacteria richness and diversity in different plant and soil compartments, Si exhibited the advantage of considerably promoting bacterial abundance in the various plant compartments. For instance, *Cetobacterium*, *Erysipelatoclostridium*, *Ruminococcus_NK4A214_group,* and *Christensenellaceae_R-7_group* were more prevalent, especially in the leaf compartment. *Cetobacterium* is a Gram-negative, non-spore-forming, pleomorphic, non-motile, and rod-shaped genus of bacteria from *Fusobacteriaceae* family. Moreover, *Cetobacterium* is a kind of *Brevibacterium* that can oxidize sugar and alcohol into acetic acid and other products, which suggests that Si may have been involved in regulating microorganisms to improve sugar metabolism in sugarcane leaves (Ramírez et al., 2018). Studies have proved that a proper amount of *Erysipelatoclostridium* and *Ruminococcaceae NK4A214* group can improve intestinal health (Zhang et al., 2020; Milosavljevic et al., 2021). *Christensenellaceae R-7 group* can regulate lipid metabolism and could reduce the incidence of obesity (Goodrich et al., 2014).

In sugarcane stems, Si treatment significantly increased *Lachnospiraceae_UCG_009*, *Allobaculum,* and *Akkermansia* compared with the other compartments. It has been documented that *Lachnospiraceae* belong to the core of gut microbiota colonizing the intestinal lumen from birth and increasing. Huang et al. (2019) employed reductive soil disinfestation (RSD), also known as anaerobic soil disinfestation or biological soil disinfestation, an environmentally sustainable approach used to mitigate soil-borne diseases. They observed that *Lachnospiraceae* was one of the prevalent bacteria in the entire sample. Tasnim et al. (2021) mentioned that *Allobaculum* is beneficial bacteria that can produce short-chain fatty acids in the intestine. Moreover, it plays an important role in anti-inflammation, protecting intestinal barrier function, regulating human metabolism and immunity directly or indirectly by increasing the content of short-chain fatty acids in intestines, especially in the process of preventing or treating metabolic diseases such as obesity, insulin resistance, and diabetes. *Akkermansia* is a new generation of probiotics and is considered the most abundant mucolytic bacteria in the intestines of healthy people. It has also the potential to protect the intestines from pathogens (Derrien et al., 2004; Belzer and de Vos, 2012). These findings further conformed that a number of microbial tend to enter plant roots system as endophytes and establish a mutual relationship with their host, which in line with previous studies (Afzal et al., 2019; Dubey et al., 2020).

In the rhizosphere soil of sugarcane, Si treatment significantly increased the abundance of *Mesorhizobium* and *Methylopila*. Studies have documented that rhizobacterial genera, e.g., *Rhizobium*, *Bradyrhizobium,* and *Mesorhizobium* are capable of promoting legumes growth and making nitrogen available to plants in metal-contaminated environments (Drinkwater et al., 2017; Shahid et al., 2021). *Methylotrophic* bacterial composition plays a crucial role in sustainable agriculture by enhancing soil fertility and health, thereby boosting plant growth promotion (PGP) and crop yield, evident by the significant increase in sugarcane traits, namely, stalk height and theoretical production (Kumar et al., 2019).

Co-occurrence interactions are environmentally essential patterns that reveal niche processes that trigger diversity maintenance within biological communities and coexistence (Williams et al., 2014; de Vries et al., 2018). However, little is known about how co-occurrence networks within these communities in different soil and plant compartments respond to agriculture practices such as fertilization, specifically Si amendment. Here, we adopted co-occurrence network to decipher potential communications of community assembly in the different soil and plant tissues. We noticed that Si amendment has the potential to increase bacterial diversity maintenance, coexistence, and plant–soil systems bacteria connections, which, in turn, had a positive growth effect on sugarcane growth compared with the CK treatment. This finding partly conformed with Zheng et al. (2018) study, wherein it was mentioned that soil management practices, namely, cover crops potentially increased bacterial community co-occurrence networks, but not within the fungal community.

A number of studies have reported that the extractable pool of metabolites has the potential to interact with the soil microbial communities (Swenson et al., 2015; Song et al., 2020). For instance, Jones et al. (2004) documented that plant exudates comprising predominantly of sugars, organic acids, and amino acids stimulated microbes’ activity in soils. Hence, it is important to have a broader understanding of the interaction between extractable pools of metabolites and microbial communities. In this study, we explored the relationships between the differential microbes and differential metabolites data generated from the different plant tissues (root, stem, and leaf) by constructing an interactive network. Generally, the analysis demonstrated that there were more negative and positive correlations than correlations in the network within the metabolites and microbes communities. However, SA demonstrated a significant positive association with a majority of bacteria in the plant tissues, including *f_micropipesaceae, Dyella, Salix Integra, Burkholderia Caballeria Paraburkholderia, Catenulispora, f_Chitinophagaceae,* and *Thermosporothrix*. We also found that d-fructose displayed a positive correlation with *Enhydrobacter* and *Akkermansia*, whereas raffinose was positively correlated with *Enhydrobacter* in the different plant tissues. While in the different soil regions, raffinose demonstrated a significant positive association with *Bradyrhizobium, Catenulispora,* and *Sphingomonas*. This finding is partly in agreement with Song et al. (2020) work. They reported that differential metabolites, such as sucrose displayed a considerable relationship with the soil bacterial members, including *Rhizobiaceae* family, *Betaproteobacteria* class, *Burkholderiales* order, *Proteobacteria* phylum, and *Ensifer* genus. We, therefore, inferred that bacteria with increased abundances and positive correlations with these mentioned metabolites above especially in the different plant tissues tended to favor the production and yield of sugarcane.

We also assessed the relationship among the differential microbes, differential metabolites, and soil properties. It was noticed that soil Si content demonstrated a considerable positive association with the bacterial communities, namely, *Conexibacter*, *OM o_IMCC26256,* and *Acidothermus*. This result is in agreement with the finding mentioned by Huang et al. (2021), who documented that the nitrogen concentration in soil and the flavonoid content in stalks were markedly associated with the soil microbiome composition.

## Conclusion

Taken together, our study demonstrated that Si utilization had a marked positive effect on sugarcane agronomic parameters and soil physicochemical properties. Moreover, the trend of bacterial richness and diversity index was soil > root > stem > leaf. It was also noticed that plant and soil compartments exhibited a modulatory effect on bacterial abundance and community compositions. Co-occurrence interactions showed that Si amendment has the potential to increase bacterial diversity maintenance, coexistence, and plant–soil systems bacteria connections, thereby increasing the functional diversity in the various plant tissues, which, in turn, could trigger positive growth effects in sugarcane. Network analysis further revealed that metabolite profiles exhibited a strong association with bacterial community structures. It was revealed that Si content had a considerable positive association with bacterial communities. This study provides comprehensive empirical evidence of the significance of Si in agriculture and illuminated on differential metabolite profiles and soil microbes relationship.

## Data availability statement

The datasets presented in this study can be found in online repositories. The names of the repository/repositories and accession number(s) can be found in the article/Supplementary material.

## Author contributions

ZY: conceptualization, data curation, funding acquisition, supervision, and project administration. ZP: data curation, formal analysis, investigation, methodology, software, visualization, and writing–review and editing. NF: formal analysis, software, writing–original draft, writing–review and editing, and methodology. YZ and FD: formal analysis and software. WL: conceptualization, funding acquisition, and resources. CH: supervision, funding acquisition, and resources. All authors contributed to the article and approved the submitted version.

## Funding

This research was funded by the National Natural Science Foundation of China (31771723), the Modern Agricultural Industry Technology System of China (CARS-170208), and the Special Foundation for Scientific and Technological Innovation of Fujian Agriculture and Forestry University (CXZX2016172 and CXZX2017349) and supported by the China Agriculture Research System of MOF and MARA.

## Conflict of interest

The authors declare that the research was conducted in the absence of any commercial or financial relationships that could be construed as a potential conflict of interest.

## Publisher’s note

All claims expressed in this article are solely those of the authors and do not necessarily represent those of their affiliated organizations, or those of the publisher, the editors and the reviewers. Any product that may be evaluated in this article, or claim that may be made by its manufacturer, is not guaranteed or endorsed by the publisher.
