# Supplementary figures and images for: Silicon fertilizer mediated structural variation and niche differentiation in the rhizosphere and endosphere bacterial microbiome and metabolites of sugarcane
Source: Front Microbiol. 2022 Sep 29;13:1009505. doi: 10.3389/fmicb.2022.1009505 (PMC9560586; doi:10.3389/fmicb.2022.1009505)

### Multy samples Rarefaction Curves

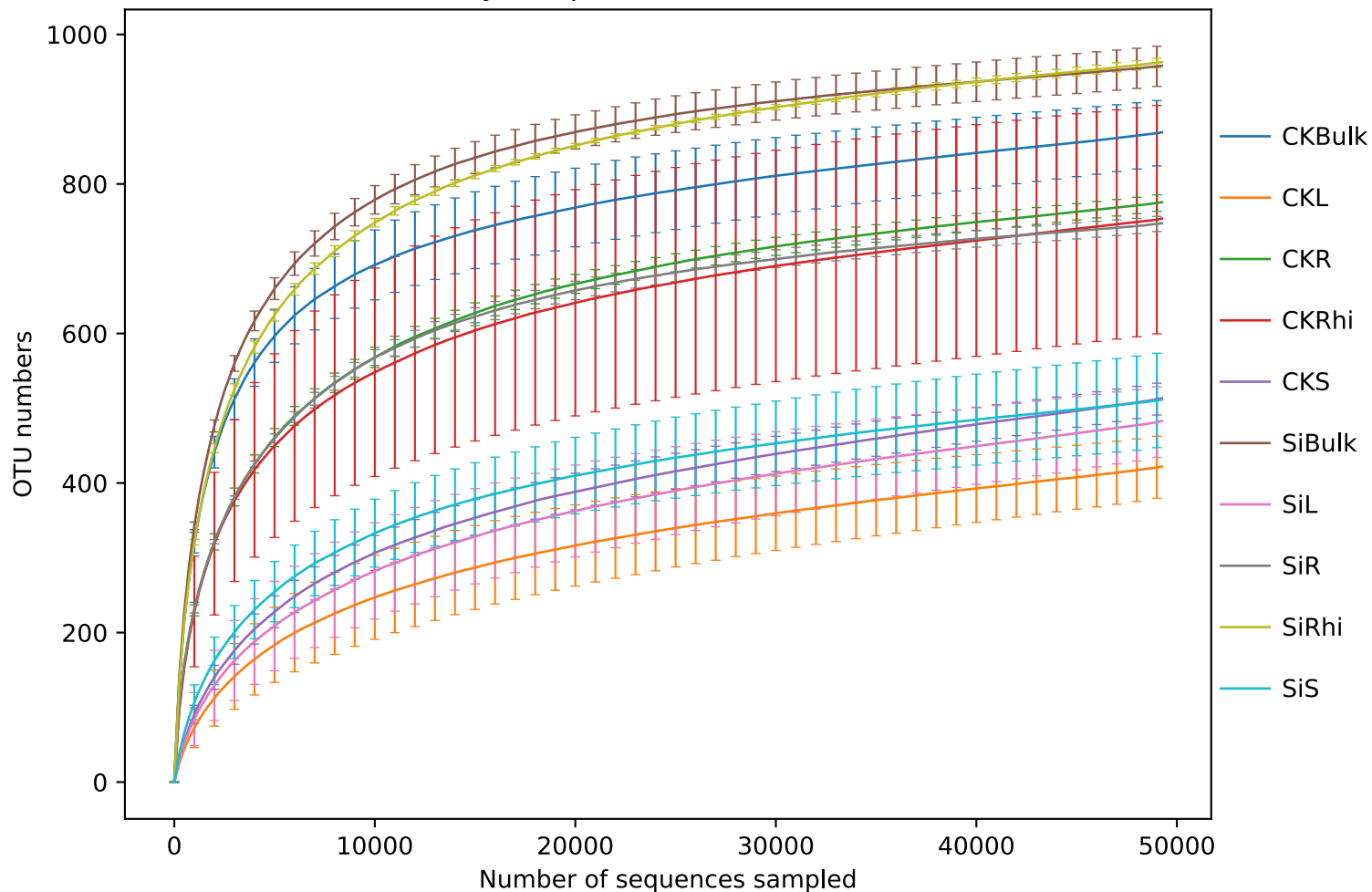

### Multy samples Shannon Curves

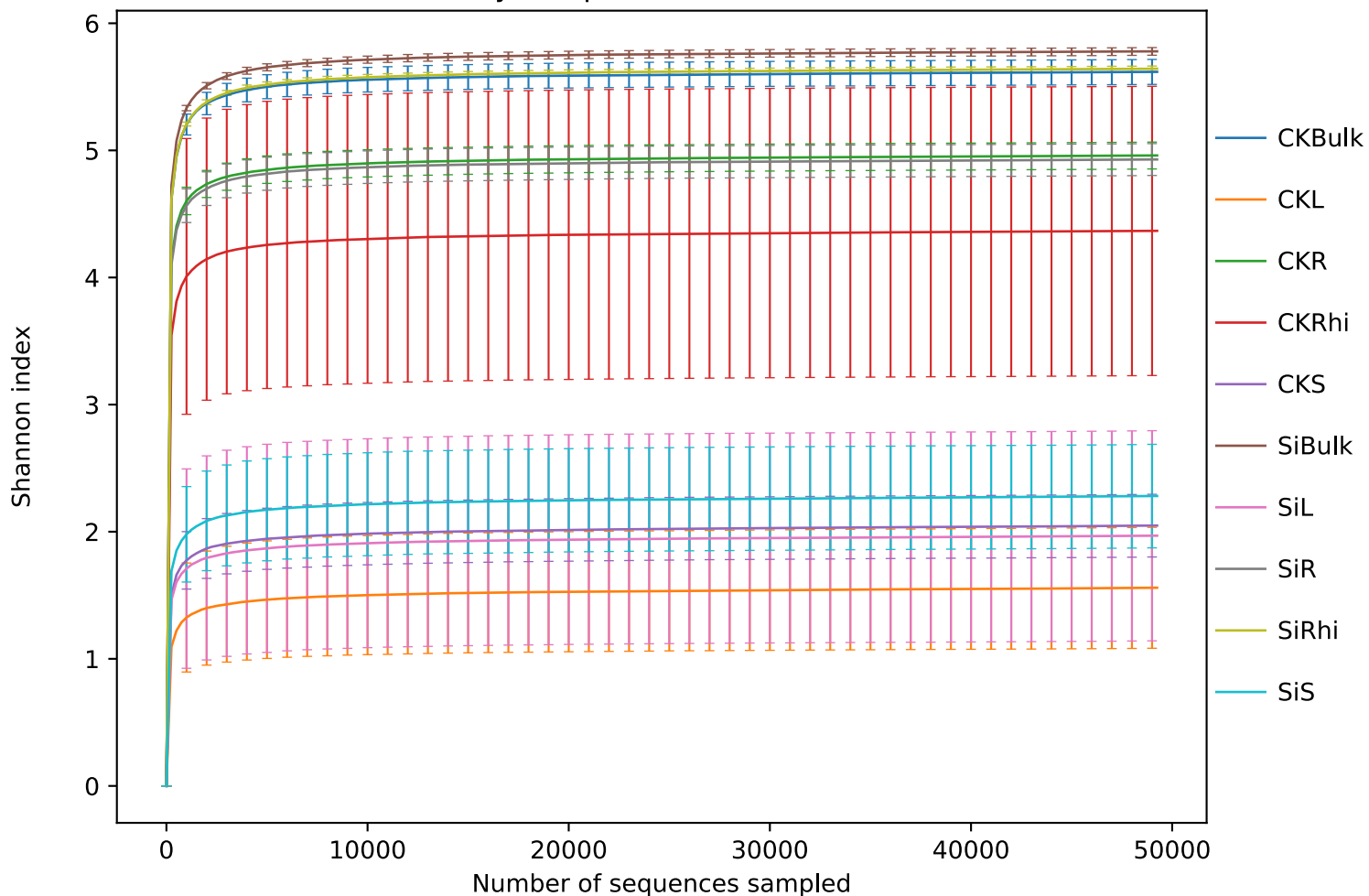

Supplement: Supplementary file 9 [file Data_Sheet_1.PDF]

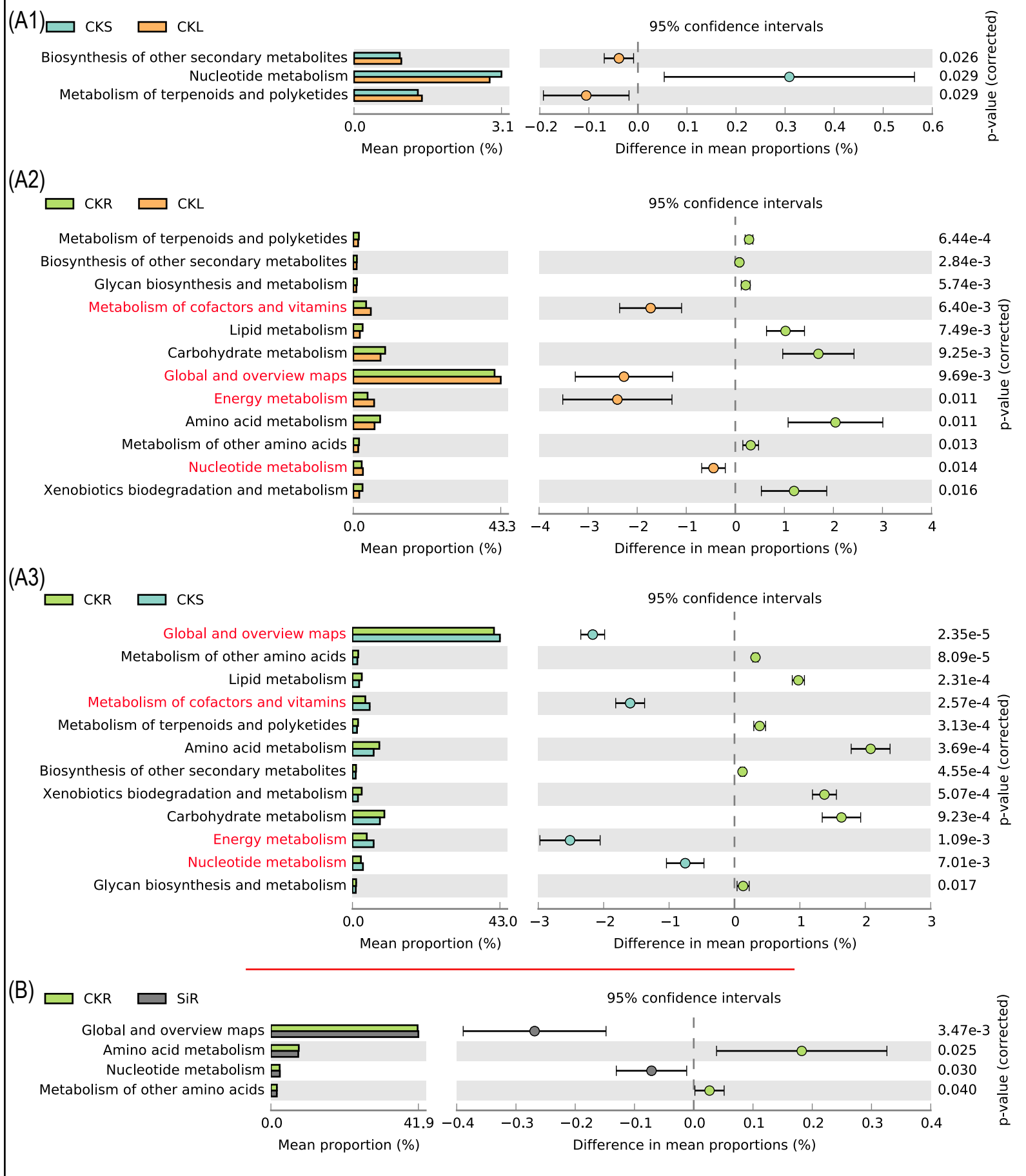

Supplement: Supplementary file 11 [file Data_Sheet_3.PDF]
