# Supplementary material for: Silicon fertilizer mediated structural variation and niche differentiation in the rhizosphere and endosphere bacterial microbiome and metabolites of sugarcane
Source: Front Microbiol. 2022 Sep 29;13:1009505. doi: 10.3389/fmicb.2022.1009505 (PMC9560586; doi:10.3389/fmicb.2022.1009505)
Supplement: Supplementary file 10 [file Data_Sheet_2.PDF]

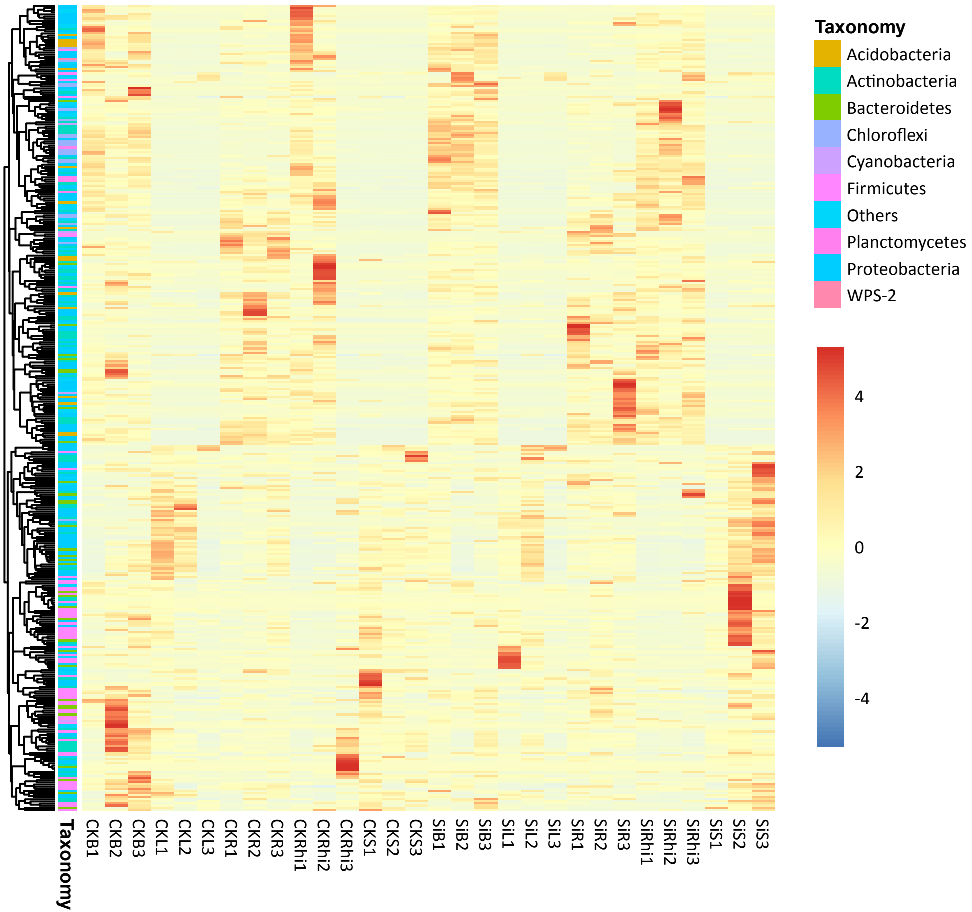

Taxonomy
Acidobacteria
Actinobacteria
Bacteroidetes
Chloroflexi
Cyanobacteria
Firmicutes
Others
Planctomycetes
Proteobacteria
WPS-2
4
2
0
-2
-4
Taxonomy
CKB1
CKB2
CKB3
CKL1
CKL2
CKL3
CKR1
CKR2
CKR3
CKRhi1
CKRhi2
CKRhi3
CKS1
CKS2
CKS3
SiB1
SiB2
SiB3
SiL1
SiL2
SiL3
SiR1
SiR2
SiR3
SiRhi1
SiRhi2
SiRhi3
SiS1
SiS2
SiS3
